# Survey of gut microbial biogeography and their functional niche in the grow-finishing swine of ordinary feeding

**DOI:** 10.3389/fmicb.2025.1530553

**Published:** 2025-03-07

**Authors:** Lili Cao, Wei Guo, Shiyu Yang, Anum Ali Ahmad, Yuntao Dong, Cen Gong, Shuoqi Wang, Xuemin Yang, Zhentao Cheng, Zhihong Yan, Weiwei Wang

**Affiliations:** ^1^Laboratory of Animal Genetics, Breeding and Reproduction in the Plateau Mountainous Region, Ministry of Education, College of Animal Science, Guizhou University, Guiyang, China; ^2^Key Laboratory of Animal Diseases and Veterinary Public Health of Guizhou Province, College of Animal Science, Guizhou University, Guiyang, China; ^3^College of Resources and Environmental Engineering, Guizhou University, Guiyang, China; ^4^Guizhou Yuhong Biotechnology Co., Ltd., Guiyang, China; ^5^The Roslin Institute and Royal (Dick) School of Veterinary Studies, University of Edinburgh, Edinburgh, United Kingdom; ^6^Institute of New Rural Development, Guizhou University, Guiyang, China

**Keywords:** swine, gut microbial biogeography, microbial diversity, microbial functional niche, KEGG enrichment analysis

## Abstract

**Background:**

Swine represent one of the most economically significant livestock worldwide, and their intestinal microbial communities are crucial for maintaining physiological development and regulating host metabolism. While extensive research has focused on the fecal microbiota of swine, investigations into microbial communities across different intestinal segments remain limited.

**Objective:**

This study aims to elucidate the intestinal microbiota of swine by analyzing luminal contents from different intestinal segments, including the duodenum, jejunum, ileum, cecum, and colon.

**Methods:**

We employed 16S rRNA sequencing to explore the diversity and structure of gut microbial biogeography, microbial functional niches, and their associated pathways.

**Results:**

Our findings reveal significantly lower microbial richness and diversity in the small intestine (duodenum, jejunum, and ileum) compared to the large intestine (cecum and colon) (*p* < 0.05). At the phylum level, Firmicutes, Actinobacteria, Proteobacteria, and Bacteroidetes were the dominant phyla, collectively accounting for over 90% of the total sequences. In the small intestine, Proteobacteria (4.76–34.2%), Actinobacteria, and Fusobacteriota were more abundant, whereas in the large intestine, Firmicutes (89.8–90.4%) was predominated. At the genus level, *Fusobacterium*, *Corynebacterium*, *Rothia*, *Bradyrhizobium*, and *Brevundimonas* were predominant in duodenum. *Romboutsia*, *Clostridium_sensu_stricto_1*, *Terrisporobacter*, and *Jeotgalicoccus* demonstrated greater abundances in the jejunum and ileum. *Oscillospiraceae_UCG-005* in the cecum and *Christensenellaceae_R-7_group* in the colon were more abundant with 16.4 and 20.2% relative abundances, respectively. The specialists detected from the duodenum to the colon were all the predominant genera in each intestinal segment with relatively higher relative abundance. For instance, *Romboutsia* (3.06–36.1%), *Clostridium_sensu_stricto_1* (5.31–18.6%), and *Terrisporobacter* (0.849–5.72%) were dominant genera and specialists in the small intestine, associated with enriched pathways of Amino acid metabolism and Lipid metabolism. Conversely, *Oscillospiraceae_UCG-005* (16.4%, 4.06%) and *Christensenellaceae_R-7_group* (5.44%, 20.2%) are predominant genera and specialists within the large intestine, linked to pathways involved in Glycan biosynthesis and metabolism pathway, as well as the Biosynthesis of other secondary metabolites.

**Conclusion:**

These highlight the importance of genus specialists compared to genus generalists. The findings provide essential data for assessing the role of the intestinal microbiome in maintaining and enhancing swine health and productivity, offering fundamental guidance for further exploration of host-microbe interaction mechanisms and regulatory pathways.

## 1 Introduction

The gut, an auxiliary metabolic organ of the host, harbors a complex community of microorganisms referred to as gut microbes or gut flora (Ahrodia et al., 2022). An increasing body of research suggests that the gut microbiota plays a vital role in sustaining physiological balance, supporting the development of the immune system, and regulating host metabolism (Cani, 2017; Gomaa, 2020; Wang et al., 2024). Swine are among the most economically valuable livestock globally, serving as vital source of meat for human consumption and as a biomedical model for human disease research (Xiao et al., 2016). Consequently, understanding the composition of gut microbiota is essential for maintaining the health and productivity of swine. To date, studies examining the intestinal microbiota of swine have predominantly focused on fecal and rectal microbiota, primarily due to the convenience of sample collection. However, the microbial flora varies across different sections of the swine intestine due to differences in anatomical structure and physiological characteristics, which results in distinct microbial compositions in each region (Li et al., 2020; Huang and Chen, 2023). Further research to decipher the key gut microbes that function in each region would be beneficial for better regulating gut microbes and improving swine production efficiency.

The animal gut hosts a highly heterogeneous and dynamically evolving microbial ecosystem predominantly composed of bacteria (Van de Vliet and Joossens, 2022). This ecosystem encompasses various microenvironments, including the duodenum, jejunum, ileum, cecum, and colon, each selectively harboring characteristic microorganisms along the longitudinal axis of the intestinal lumen (Tropini et al., 2017). Different sections and microenvironments of the gut exhibit distinct physical and biochemical conditions, such as pH, oxygen concentration, chyme flow, peristalsis rates, and nutrient availability (Cremer et al., 2017; Zeng et al., 2022; Ng et al., 2023). The small intestine, comprising the duodenum, jejunum, and ileum, is dominated by fast-growing parthenogenetic anaerobes such as *Streptococcus*, *Lactobacillus*, and *Escherichia-Shigella*. These microbes are primarily involved in the digestion and absorption of various nutrients, including amino acids, proteins, lipids, some oligosaccharides, and monosaccharides. In contrast, the large intestine harbors a substantial number of saccharolytic anaerobes, such as *Prevotella*, *Clostridium*, and *Bacteroides* (Holman et al., 2017; Zhang L. et al., 2018), which are involved in the degradation and absorption of nutrients like resistant starch, lignin, and insoluble cellulose, which are indigestible in the small intestine (Wang H. et al., 2020). These observations underscore the importance of considering spatial distribution when describing intestinal bacteria and highlight the need to elucidate the biogeography of animal gut microbial communities. Furthermore, analyzing the diversity and biogeographic patterns of bacterial communities would provide insights into the deeper ecological processes and mechanisms that underpin and sustain bacterial diversity and ecosystem functioning (Hanson et al., 2012; Wan et al., 2023). Although our comprehension of ecosystems largely relies on dominant species, enhanced predictive capabilities can also be achieved by systematically differentiating between microbial generalists and specialists. Whether a microbe qualifies as a generalist or specialist typically depends on its ecotype width, which describes the range of resources, habitats, or environments it utilizes (Zhou and Ning, 2017). This distinction underscores the need to address gaps in our understanding of gut microbiome ecology.

The feed consumed during the grow-finishing stage of pigs accounts for 70% to 75% of the entire feeding period (Pierozan et al., 2016). If the utilization rate of feed by pigs is improved during this stage, the production efficiency will be improved and the production cost will be reduced. The role of intestinal flora in promoting intestinal maturation, regulating the immune system, and improving host health and growth performance has received more attention in the swine industry. This study aimed to comprehensively understand the spatial and ecological patterns of microbial communities in the gut of healthy grow-finishing swine using high-throughput sequencing, focusing on the composition, diversity, ecological niche breadth, and function of microorganisms in five intestinal regions, i.e., the duodenum, jejunum, ileum, cecum, and colon. The findings of this study will provide crucial data for evaluating gut microbes essential for maintaining and promoting pig health and production, while also offering guidance for further exploration of host-microbe interaction mechanisms and associated pathways.

## 2 Materials and methods

### 2.1 Animals, feed, and sample collection

Six castrated male Sanyuan (Long White Pig, Large White Pig, Jinhua Pig cross) hybrid pigs, approximately 7 months old and weighing around 110 kg, were used in this study. The animals were obtained from the Kaiyangtai Agricultural Pig Factory in Guizhou Province. All pigs were healthy, free from gastrointestinal diseases, and had not been exposed to antibiotics prior to the experimental trial. The experimental pigs were fed three times daily (09:00, 15:00, and 20:00) and were provided with *ad libitum* access to both feed (Supplementary Table S1) and water at all times. All experimental pigs were housed in a well-ventilated facility with controlled humidity (61 ± 6%) and a temperature range of 22–26°C. After 2 months’ feeding trial, the animals were humanely killed, and the intestinal contents from the duodenum, jejunum, ileum, cecum, and colon were collected and rapidly frozen in liquid nitrogen. Subsequently, the samples were transported to the laboratory and stored at −*80*°C.

### 2.2 DNA extraction and 16S rRNA amplicon sequencing

Following the manufacturer’s instructions, the genomic DNA was extracted from intestinal contents using a Bacterial DNA Isolation Kit (Foregene, Chengdu, China). The purity and concentration of the DNA were determined using a Nanodrop 2000 spectrophotometer (Thermo Fisher Scientific, US), while the integrity of the DNA was assessed through agarose gel electrophoresis. The V3–V4 region of the 16S rRNA gene was PCR amplified using the sequencing primer pair 338F (5′-ACTCCTACGGGAGGCAGCAG-3′) and 806R (5′-GGACTACHVGGGTWTCTAAT-3′). The PCR products from the same samples were combined and visualized via 2% agarose gel electrophoresis. The amplicons were purified using the Axy Prep DNA Gel Extraction Kit, and the purified products were subsequently detected and quantified using the Quantus™ Fluorometer. Sequencing libraries were prepared using the NEXTFLEX Rapid DNA-Seq Kit and then sequenced on the Illumina MiSeq PE300 platform.

### 2.3 Taxonomy, differential abundance, and LEfSe analysis

The reads from each sample were spliced using FLASH software (version 1.2.11)^1^ (Magoč and Salzberg, 2011). Cleaned reads were then clustered into operational taxonomic units (OTUs) at 97% similarity using UPARSE software (Edgar, 2013), which enabled the calculation of the ecological niche breadth for each OTU (Jiao et al., 2020; Mo et al., 2021; Aslani et al., 2022). Meanwhile, taxonomic classification at the species level was determined by comparing the OTU tables against the Silva 16S rDNA database (v138) using the RDP classifier (version 2.2)^2^ (Wang and Cole, 2024). Subsequently, Mothur software was used to calculate the alpha diversity indices, which are coverage, Sobs, Ace, Chao 1, Shannon, and Simpson index (Douglas et al., 2020). The coverage index was employed to assess the community coverage across all samples, while the Sobs index was defined as the number of species observed in the sample (i.e., OTU count). The Ace and Chao 1 indices were calculated to evaluate community richness, and the Shannon and Simpson indices were used to compare community diversity. Based on the Bray-Curtis distance metric, Beta diversity of bacterial community was compared using principal coordinate analysis (PCoA). Taxa with a relative abundance of less than 0.01 in all samples were grouped as “Others.”

Linear discriminant analysis effect size (LEfSe) analyses were performed online^3^ to identify bacterial taxa exhibiting significantly different abundances among the different at each taxonomic level (LDA threshold = 4, *p* < 0.05) (Wang L. et al., 2020).

### 2.4 Microbial niche breadth and metabolic pathway analysis

Ecological niche breadth is a critical indicator in elucidating the diversity and co-occurrence patterns of microbes (Jiao et al., 2020). Communities with a wider ecological niche are typically more metabolically versatile (Wu et al., 2018). To assess the community-level niche breadth, the ecological niche breadth of all community members was calculated using Levins’ niche breadth index in the “spaa” package (Levins, 1968; Zhang, 2013; Li et al., 2021). The microbial occurrences were estimated by performing 1,000 permutations using the “EcolUtils” package (Salazar, 2015). Taxa with observed occurrences falling below the lower 95% confidence interval were identified as specialists, while those with observed occurrences exceeding the upper 95% confidence interval were classified as habitat generalists. The remaining taxa were categorized as non-significant (Zhang J. et al., 2018).

Functional predictions of bacterial communities were performed using the PICRUSt2 software (v2.2.0) (Douglas et al., 2018; Douglas et al., 2020). Bacterial functions were annotated according to the Kyoto Encyclopedia of Genes and Genomes (KEGG) database, and their metabolic pathways analyzed using STAMP software (Parks et al., 2014).

### 2.5 Statistical analysis

All data are presented as mean ± SEM (standard error of the mean). Duncan’s multiple comparison test was applied to compare the alpha diversity of the microbial communities. The Kruskal-Wallis test was used to assess the differences in microbial communities across the intestinal segments. Orthogonal polynomial contrasts were applied using SAS 9.2 software (SAS Institute, Cary, NC) to evaluate whether the responses to the transition from the small intestine to the large intestine followed a linear or quadratic pattern. Statistical *p*-value less than 0.05 represents significance, while *p*-value more than 0.05 and less than 0.10 suggests tendencies.

## 3 Results

### 3.1 Sequencing data and diversity analysis of the swine gut microbial community

**FIGURE 1:**
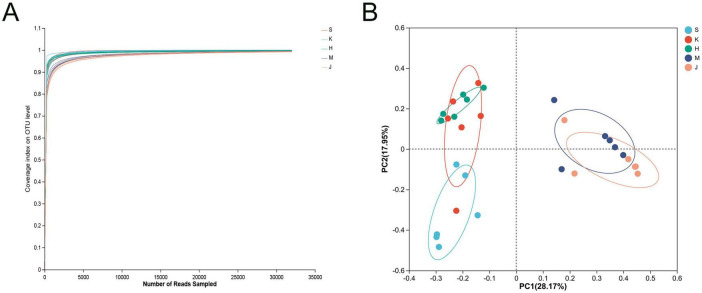
**(A)** Coverage curve of microbial community and **(B)** principal coordinate analysis (PCoA) based on the Bray distance matrix. S, Duodenum; K, Jejunum; H, Ileum; M, Cecum; J, Colon.

On average, 69, 628 reads were generated for each sample, with an average length of 411 bp, obtained after quality filtering using UPARSE software. The coverage curve for the microbial community reached a plateau, indicating sufficient sequencing depth (Figure 1A). PCoA demonstrated the clustering of the microbial community from five intestinal segments into two groups. The microbial community structure of the small intestine (duodenum, jejunum, and ileum) was distinct from that of the large intestine (the cecum and colon), resulting in different clusters. Furthermore, the microbial community structure in the duodenum clustered separately from that in the ileum (Figure 1B).

The alpha diversity, as indicated by Sobs, Ace, Chao 1, and Shannon indices, showed a linear increase from the duodenum to the colon (*p* < 0.01); however, there were no differences observed between the cecum and colon (*p* > 0.05) (Table 1). The Sobs, Ace, Chao 1, and Shannon indices of the large intestine (cecum and colon) were higher compared to the small intestine (duodenum, jejunum, and ileum) (*p* < 0.001). The highest microbial diversity and abundance were observed in the cecum and colon, while the lowest were found in the jejunum.

**TABLE 1:** The alpha diversity indices of intestinal bacterial community across different segments.

Diversity	Duodenum	Jejunum	Ileum	Cecum	Colon	SEM	*p*-values		
							**Different segments**	**Linear**	**Quadratic**
Sobs	253^c^	314^c^	343^c^	765^b^	876^a^	46.3	<0.001	<0.001	<0.001
Ace	273^c^	386^bc^	515^b^	989^a^	1101^a^	69.4	<0.001	<0.001	<0.01
Chao 1	275^d^	395^cd^	457^c^	944^b^	1070^a^	58.6	<0.001	<0.001	<0.001
Shannon	3.60^a^	2.60^b^	2.92^b^	3.89^a^	4.26^a^	0.306	<0.001	<0.01	<0.05
Simpson	0.063^b^	0.257^a^	0.142^ab^	0.083^b^	0.046^b^	0.059	<0.01	0.112	0.088

Superscripts with different lower case letters indicate significant differences in the means of the samples (*p* < 0.05).

### 3.2 Microbial composition of different intestinal segments of swine

**FIGURE 2:**
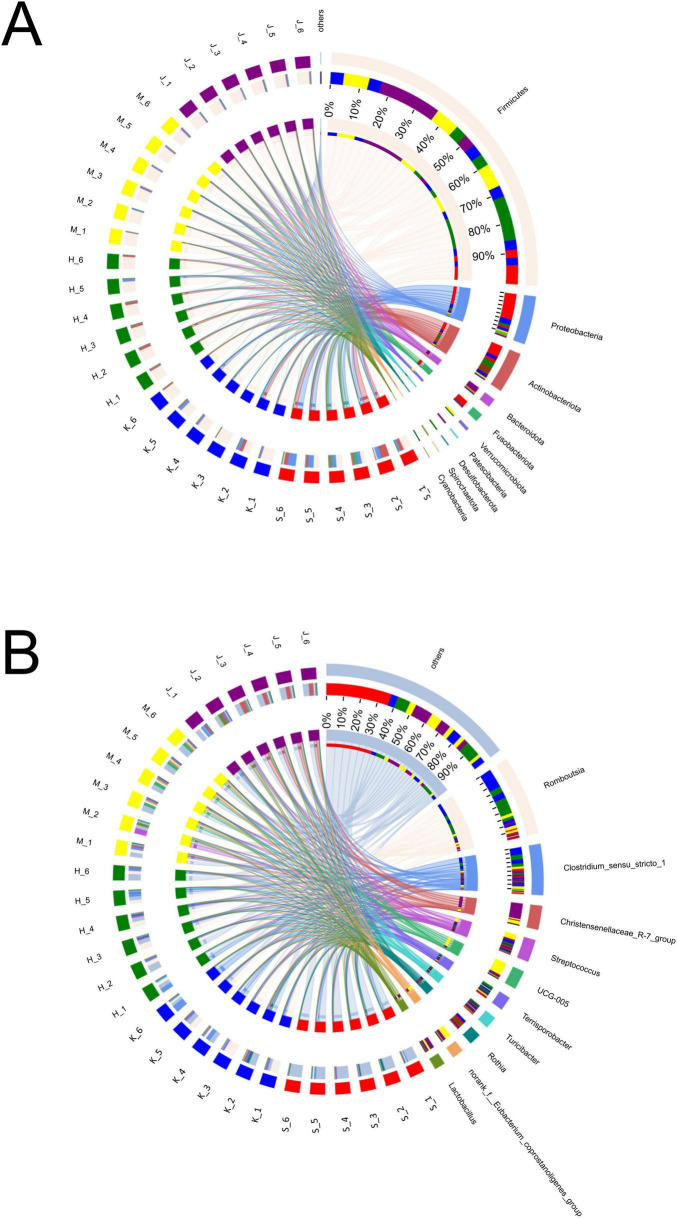
Composition and relative abundance of bacterial community at phyla **(A)** and genera **(B)** levels across different intestinal segments.

In this study, nine phyla were detected, with Firmicutes, Proteobacteria, Actinobacteriota, Bacteroidota, and Fusobacteriota identified as the dominant groups, collectively comprising over 90% of the relative abundance (Figure 2A; Table 2). The phyla Proteobacteria and Actinobacteriota predominated in the small intestine, whereas Firmicutes and Bacteroidota were more abundant in the large intestine (*p* < 0.01). Firmicutes showed the relatively high relative abundance (32.5–90.4%), with a linear increase from the duodenum to the colon (*p* < 0.001). Specifically, Firmicutes displayed the lowest relative abundance (32.5%) in the duodenum and significantly higher relative abundance in both the cecum (89.8%) and colon (90.4%). Proteobacteria and Actinobacteriota were abundant in the small intestine (duodenum: 34.2 and 23.3%, jejunum: 10.4 and 7.79%). Fusobacteriota was more abundant in the duodenum (7.68%) than in other intestinal segments. In the large intestine, Bacteroidota (colon: 7.06%) was dominant.

**TABLE 2:** The relative abundance of bacterial community at the phylum level across different intestinal segments.

Phylum (%)	Duodenum	Jejunum	Ileum	Cecum	Colon	SEM	*p*-values		
							**K-W^1^**	**Linear**	**Quadratic**
Firmicutes	32.5^b^	80.6^ab^	82.8^a^	89.8^a^	90.4^a^	6.87	<0.001	<0.001	<0.001
Proteobacteria	34.2^a^	10.4^ab^	4.76^abc^	3.57^b^	0.503^c^	4.83	<0.001	<0.001	<0.01
Actinobacteriota	23.3^a^	7.79^ab^	11.5^a^	1.87^b^	0.704^b^	5.44	<0.001	<0.001	0.295
Fusobacteriota	7.68^a^	0.568^ab^	0.555^ab^	0.079^b^	0.018^b^	2.78	<0.05	<0.05	<0.076
Bacteroidota	1.06^ab^	0.165^b^	0.137^b^	1.27^ab^	7.06^a^	0.463	<0.001	<0.001	<0.001
Others^2^	1.21^ab^	0.406^bc^	0.265^c^	3.40^a^	1.31^a^	0.673	<0.001	<0.05	0.688

^1^Results of Kruskal-Wallis test.

^2^The taxa exhibiting a relative abundance of less than 0.01 in all samples were grouped as “Others.” Superscripts with different lower case letters indicate significant differences in the means of the samples (*p* < 0.05).

Genera *Fusobacterium*, *Corynebacterium*, *Rothia*, *Bradyrhizobium*, *Brevundimonas*, *Paracoccus*, *Pseudomonas*, *Dietzia*, *Acinetobacter*, *Sphingomonas*, *Peptostreptococcus*, *Rhodococcus*, *Microbacterium*, *Gemella*, and *Aquabacterium* exhibited higher relative abundance in the duodenum (Figure 2B; Table 3). Among them, the relative abundance of genera *Fusobacterium* (7.66%), *Corynebacterium* (6.43%), *Rothia* (5.71%), *Bradyrhizobium* (5.24%), and *Brevundimonas* (5.21%) were more than 5% in the duodenum and showed a decreasing trend from the duodenum to the rest of the intestinal sections (*p* < 0.05). *Romboutsia*, *Clostridium_sensu_stricto_1*, *Escherichia-Shigella*, *norank_f_Alcaligenaceae* were highly abundant in the jejunum. *Terrisporobacter* (5.72%), *Jeotgalicoccus* (5.57%), *Salinicoccus* (4.24%), *Yaniella* (1.97%), and *norank_f_Bacillaceae* (1.47%) were higher in ileum than in other segments (*p* < 0.05). Both genera *Romboutsia* (36.1%, 31.1%) and *Clostridium_sensu_stricto_1* (18.6%, 15.1%) demonstrated greater abundances in the jejunum and ileum, respectively. In the cecum, genera *Oscillospiraceae_UCG-005* (16.4%), *norank_f_Eubacterium_coprostanoligenes_group* (8.28%), *Family_XIII_AD3011_group* (2.89%), *Akkermansia* (2.69%), *norank_f_norank_o_RF39* (2.16%), and *norank_f_Erysipelotrichaceae* (1.72%) were more abundant. While *Christensenellaceae_R-7_group* (20.2%), *Oscillospiraceae_UCG-002* (6.69%), *unclassified_f_Lachnospiraceae* (4.09%), *norank_f_Muribaculacea* (2.67%), *NK4A214_group* (1.83%), *norank_f_norank_o_Clostridia_UCG-014* (1.92%), *Ruminococcus* (1.31%), *Lachnospiraceae_NK4A136_group* (1.08%), and *Monoglobus* (1.03%) demonstrated greater abundances in the colon. The genus *Christensenellaceae_R-7_group* (20.2%) exhibited a linear increase throughout the entire intestinal tract, with the colon being its primary site. The genera *Turicibacter*, *Streptococcus*, and *Lactobacillus* did not show any significant difference in the relative abundance between the small and large intestines.

**TABLE 3:** The relative abundance of significant bacterial genera across different intestinal segments.

Genus (%)	Duodenum	Jejunum	Ileum	Cecum	Colon	SEM	*p*-values		
							**K-W^1^**	**Linear**	**Quadratic**
*Fusobacterium*	7.66^a^	0.568^ab^	0.555^ab^	0.079^ab^	0.018^b^	2.79	<0.05	<0.05	0.077
*Corynebacterium*	6.43^a^	1.26^ab^	2.15^a^	0.203^b^	0.139^b^	1.83	<0.01	<0.01	0.139
*Rothia*	5.71^a^	4.16^a^	3.12^ab^	0.023^bc^	0.010^c^	2.48	<0.001	<0.01	0.879
*Bradyrhizobium*	5.24^a^	0.928^a^	0.653^a^	0^b^	0^b^	1.07	<0.001	<0.001	<0.01
*Brevundimonas*	5.21^a^	0.488^a^	0.461^a^	0^b^	0^b^	1.90	<0.001	<0.05	0.086
*Paracoccus*	4.56^a^	0.461^a^	0.002^b^	0.005^b^	0.003^b^	2.61	<0.01	0.114	0.222
*Pseudomonas*	4.54^a^	1.06^a^	0.706^a^	0^b^	0.001^b^	0.979	<0.001	<0.001	<0.05
*Dietzia*	3.61^a^	1.35^ab^	2.50^a^	0.233^ab^	0.011^b^	1.28	<0.01	<0.01	0.846
*Acinetobacter*	2.68^a^	0.666^a^	0.287^a^	0.001^b^	0.003^b^	0.576	<0.001	<0.001	<0.05
*Sphingomonas*	2.30^a^	0.508^a^	0.492^a^	0^b^	0^b^	0.460	<0.001	<0.001	<0.05
*Peptostreptococcus*	2.01^a^	0.364^a^	0.524^a^	0.032^ab^	0^b^	0.946	<0.01	<0.05	0.312
*Rhodococcus*	2.72^a^	0.146^a^	0.008^ab^	0.002^b^	0.003^b^	1.41	<0.01	0.089	0.169
*Microbacterium*	1.87^a^	0.017^ab^	0.003^b^	0.001^b^	0.002^b^	0.629	<0.01	<0.05	<0.05
*Gemella*	1.86^a^	1.41^a^	0.732^a^	0.008^b^	0.001^b^	0.651	<0.001	<0.01	0.629
*Aquabacterium*	1.21^a^	0.383^a^	0.264^ab^	0.001^bc^	0^c^	0.255	<0.001	<0.001	<0.05
*Romboutsia*	3.06^b^	36.1^a^	31.1^a^	10.9^ab^	6.71^ab^	8.31	<0.01	0.347	<0.001
*Clostridium_sensu_stricto_1*	5.31	18.6	15.1	7.62	9.55	6.03	0.164	0.855	0.107
*Escherichia-Shigella*	0.306^b^	3.12^a^	0.742^b^	2.82^ab^	0.322^b^	1.33	<0.05	0.930	0.091
*norank_f_Alcaligenaceae*	0.247	1.28	0.002	0.001	0	0.816	0.295	0.342	0.719
*Terrisporobacter*	0.849^b^	4.37^a^	5.72^a^	3.13^ab^	1.92^ab^	1.18	<0.01	0.737	<0.001
*Jeotgalicoccus*	2.66^ab^	0.528^ab^	5.57^a^	0.402^ab^	0.004^b^	1.93	<0.01	0.220	0.199
*Salinicoccus*	0.328^ab^	0.070^ab^	4.24^a^	0.413^ab^	0^b^	1.32	<0.01	1	<0.05
*Turicibacter*	1.61	2.87	3.86	1.34	3.53	1.51	0.098	0.502	0.683
*Yaniella*	0.299^ab^	0.045^ab^	1.97^a^	0.251^ab^	0.001^b^	0.540	<0.01	0.749	<0.05
*norank_f_Bacillaceae*	0.093^ab^	0.043^ab^	1.47^a^	0.077^ab^	0^b^	0.543	<0.01	0.901	0.056
*Oscillospiraceae_UCG-005*	0.007^b^	0.058^b^	0.011^b^	16.4^a^	4.06^a^	2.78	<0.001	<0.001	0.267
*norank_f_Eubacterium_* *coprostanoligenes_group*	0.068^b^	0.023^b^	0.036^b^	8.28^a^	4.14^a^	1.21	<0.001	<0.001	0.990
*Streptococcus*	3.16	5.12	5.02	6.88	4.42	4.84	0.203	0.696	0.596
*Family_XIII_AD3011_group*	0.025^b^	0.061^b^	0.047^b^	2.89^a^	2.11^a^	0.391	<0.001	<0.001	0.243
*Akkermansia*	0.008^bc^	0.002^c^	0.001^c^	2.69^a^	0.097^ab^	0.639	<0.001	0.056	0.155
*norank_f_norank_o_RF39*	0.030^b^	0.024^b^	0.032^b^	2.16^a^	0.735^a^	0.376	<0.001	<0.001	0.477
*norank_f_Erysipelotrichaceae*	1.09^ab^	1.37^ab^	0.241^b^	1.72^a^	0.542^ab^	0.475	<0.05	0.497	0.805
*Christensenellaceae_R-7_group*	0.090^b^	0.115^b^	0.146^b^	5.44^a^	20.2^a^	3.38	<0.001	<0.001	<0.001
*Oscillospiraceae_UCG-002*	0.016^b^	0.004^b^	0.011^b^	1.96^a^	6.69^a^	0.871	<0.001	<0.001	<0.001
*Lactobacillus*	0.693	1.49	0.760	2.96	5.62	3.55	0.902	0.166	0.485
*unclassified_f_Lachnospiraceae*	0.727^b^	1.59^ab^	0.747^b^	2.65^ab^	4.09^a^	0.825	<0.01	<0.001	0.086
*norank_f_Muribaculacea*	0.004^b^	0.001^b^	0.002^b^	0.307^a^	2.67^a^	0.431	<0.001	<0.001	<0.001
*NK4A214_group*	0.048^b^	0.033^b^	0.019^b^	1.41^a^	1.83^a^	0.172	<0.001	<0.001	<0.001
*norank_f_norank_o_* *Clostridia_UCG-014*	0.083^b^	0.041^b^	0.044^b^	1.01^a^	1.92^a^	0.814	<0.001	<0.05	0.197
*Ruminococcus*	0.087^b^	0.157^ab^	0.099^b^	0.177^ab^	1.31^a^	0.195	<0.01	<0.001	<0.001
*Lachnospiraceae_NK4A136* *_group*	0.037^bc^	0.089^abc^	0.022^c^	0.431^ab^	1.08^a^	0.201	<0.001	<0.001	<0.01
*Monoglobus*	0^b^	0.009^b^	0.001^b^	0.401^b^	1.03^a^	0.146	<0.001	<0.001	<0.001
Others^2^	21.4^a^	9.11^b^	10.6^b^	14.6^ab^	15.3^ab^	2.86	<0.01	0.296	<0.001

^1^Results of Kruskal-Wallis test.

^2^The taxa exhibiting a relative abundance of less than 0.01 in all samples were grouped as “Others.” Superscripts with different lower case letters indicate significant differences in the means of the samples (*p* < 0.05).

### 3.3 LEfSe analysis and microbial niche in the community

**FIGURE 3:**
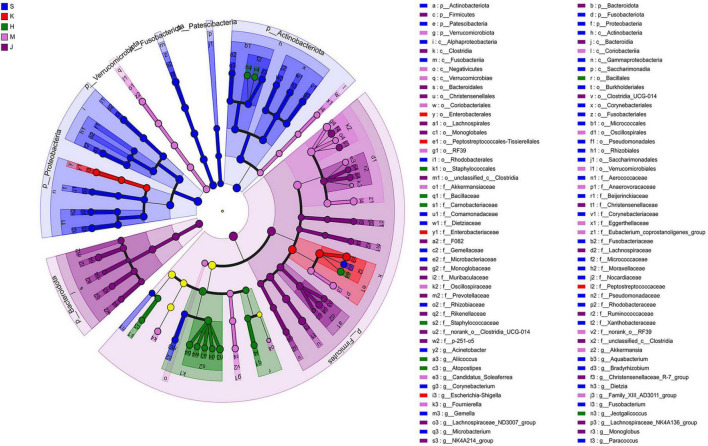
Linear discriminant analysis effect size (LEfSe) cladogram showing differentially abundant taxa across different intestinal segments. LDA scores were set to 4.0. S, Duodenum; K, Jejunum; H, Ileum; M, Cecum; J, Colon.

**FIGURE 4:**
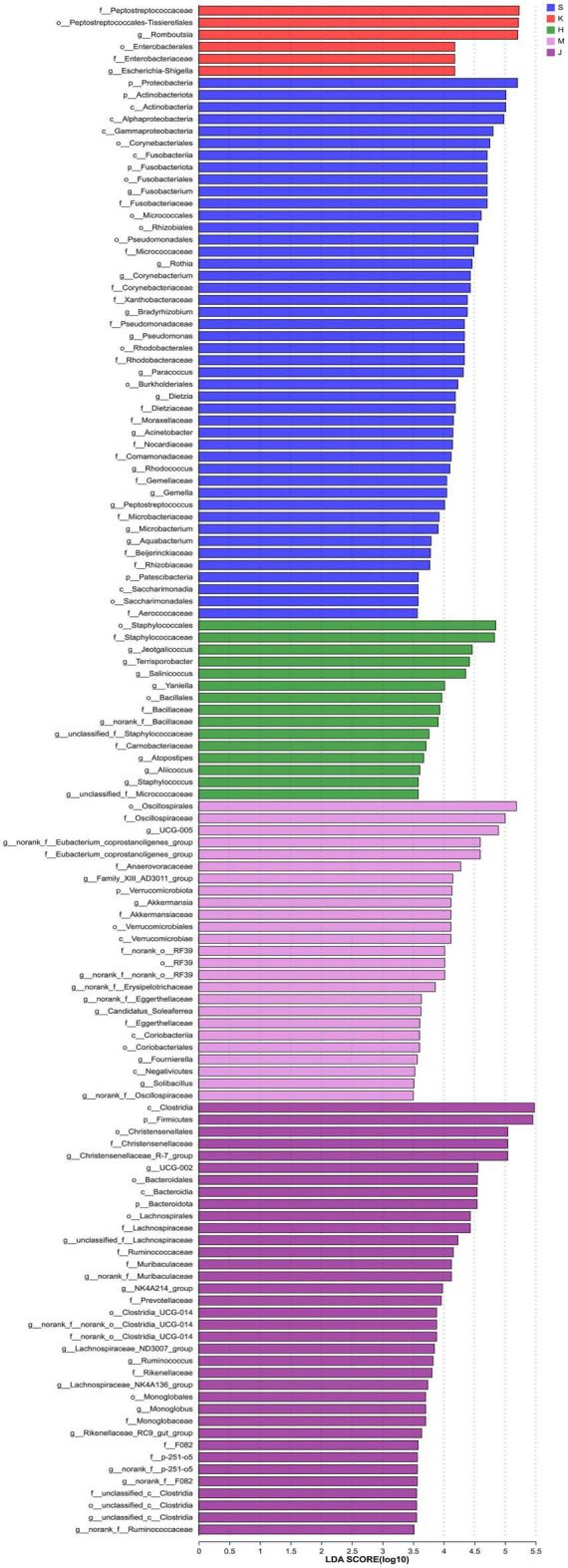
LEfSe histogram of differentially abundant taxa across intestinal segments. (LDA scores = 4.0). S, Duodenum; K, Jejunum; H, Ileum; M, Cecum; J, Colon.

The LEfSe analysis revealed that the differentially abundant OTUs predominantly belonged to the phyla Firmicutes, Proteobacteria, Actinobacteriota, Bacteroidota, Fusobacteriota, and Verrucomicrobia (Figures 3, 4). Genera from the phylum Firmicutes including *Peptostreptococcus* and *Gemella*, and those from the phylum Actinobacteriota, such as *Corynebacterium*, *Rothia*, *Dietzia*, and *Rhodococcus*, exhibited significantly higher abundances in the duodenum. Additionally, members of the Proteobacteria, including *Pseudomonas*, *Rhodobacter*, *Acinetobacter*, and *Aquabacterium*, as well as Fusobacteriota such as *Fusobacterium* were also observed. In the jejunum, genera from the phylum Firmicutes, including *Romboutsia* and Peptostreptococcaceae, and genera from the phylum Proteobacteria, including *Escherichia-Shigella* and Enterobacteriaceae were observed. In the ileum, genera *Terrisporobacter*, *Jeotgalicoccus*, *Salinicoccus*, *unclassified_f_Staphylococcaceae*, *Atopostipes*, *Aliicoccus*, *Staphylococcus* from the phylum Firmicutes, as well as genera *Yaniella* and *unclassified_f_Micrococcaceae* from the phylum Actinobacteriota, were recorded. The cecum harbored a distinct population of microorganisms from the phylum Firmicutes, including *UCG-005*, *norank_f _Eubacterium_coprostanoligenes_group*, *norank_f_norank_o_ RF39*, *Family_XIII_AD3011_group*, *norank_f_Erysipelotrichaceae*, *Candidatus_Soleaferrea*, *Fournierella*, *norank_f_Oscillospiraceae*, *unclassified_f_Lachnospiraceae*, and the genus *Akkermansia* from the phylum Verrucomicrobiota. Furthermore, the genus *norank_f_Eggerthellaceae* from the phylum Actinobacteriota and the genus *Solimonas* from the phylum Proteobacteria were also observed. In the colon, the significantly abundant genera included *Christensenellaceae_R-7_group*, *Oscillospiraceae_UCG-002*, *unclassified_f_Lachnospiraceae*, *NK4A214_group*, *Rumino coccus*, *norank_f_norank_o_Clostridia_UCG-014*, *Monoglobus*, *Lachnospiraceae_NK4A136_group*, *Lachnospiraceae_ND3007_ group*, *unclassified_c_Clostridia*, and *unclassified_Ruminococcaceae* from the phylum Firmicutes, alongside genera such as *norank_f_Muribaculaceae*, *norank_f_F082*, *Rikenellaceae_RC9_ gut_group*, and *norank_f_p-251-o5* from the phylum Bacteroidota (Figure 4).

**FIGURE 5:**
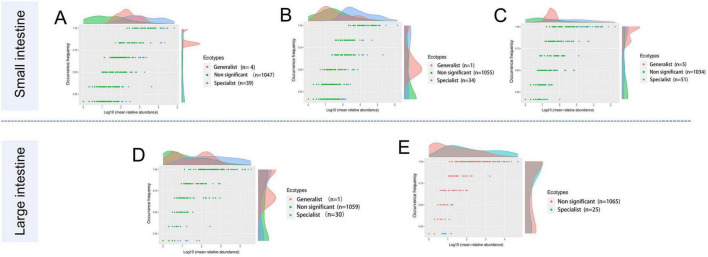
The occurrence of the microbes reflects differences in niche breadth and specialization in unique habitats. Each dot represents one genus with different colors indicating generalist, specialist, or non-significant (neutralist) taxa. **(A)** Duodenum, **(B)** Jejunum, **(C)** Ileum, **(D)** Cecum, and **(E)** Colon.

Generalists and specialists exhibited distinct adaptive capacities within microbial communities. The count of specialists identified in the intestinal ecosystem at the genus level surpassed that of generalists. Furthermore, a greater diversity of both generalists and specialists was noted in the small intestine compared to the large intestine. The abundance of specific taxa was observed along the longitudinal axis of the intestine in distinct microenvironments. The specialists detected in the duodenum included *Corynebacterium* (6.43%), *Jeotgalicoccus* (2.66%), *Paracoccus* (4.56%), *Rhodococcus* (2.72%), *Rothia* (5.71%), *Sphingomonas* (2.30%), *Streptococcus* (3.16%), *Bradyrhizobium* (5.24%), and *Brevundimonas* (5.21%). In the jejunum, *Escherichia-Shigella* (3.12%), *Terrisporobacter* (4.37%), *Clostridium_sensu_stricto_1* (18.6%), and *Romboutsia* (36.1%) were identified as specialists. Genera such as *Jeotgalicoccus* (5.57%), *norank_f_Bacillaceae* (1.47%), and *Salinicoccus* (4.24%) were recognized as specialists in the ileum. In the cecum, specialists included *Akkermansia* (2.69%), *Clostridium_sensu_stricto_1* (7.62%), and *Oscillospiraceae_UCG-005* (16.4%), while in the colon, *Christensenellaceae_R-7_group* (20.2%), *Lactobacillus* (5.62%), *Turicibacter* (3.53%), and *norank_f_norank_o_Clostridia_UCG-014* (1.92%) were identified as specialists (Figure 5; Table 4).

**TABLE 4:** Comparison of specialization in unique habitats (MRA^a^ > 1%).

Group	Generalist (MRA > 1%)	The only specialist (MRA > 1%)	Common specialist (MRA > 1%)
Duodenum	*Family_XIII_AD3011_group*, *Methylocella*, *UCG-008*, *norank_f_norank_o_RF39*	*Corynebacterium*, *Jeotgalicoccus*, *Paracoccus*, *Rhodococcus*, *Rothia*, *Sphingomonas*, *Streptococcus*	*Bradyrhizobium*, *Brevundimonas*, *Clostridium_sensu_stricto_1*, *Fusobacterium*, *Pseudomonas*, *Romboutsia*
Jejunum	*P3OB-42*	*Escherichia-Shigella*, *Microbacterium*, *Peptostreptococcus*, *Terrisporobacter*	
Ileum	*Anaerococcus*, *Ignavigranum*, *Kocuria*, *Marvinbryantia*, *norank_f_Oscillospiraceae*	*Jeotgalicoccus*, *Rothia*, *Salinicoccus*, *Yaniella*, *norank_f_Bacillaceae*, *unclassified_f_Staphylococcaceae*	*Romboutsia*, *Streptococcus*
Cecum	**–**	*Akkermansia*, *Lactobacillus*, *Clostridium_sensu_stricto_1*, *Terrisporobacter*, *Turicibacter*, *Oscillospiraceae_UCG-005*, *unclassified_f_Lachnospiraceae*	
Colon	**–**	*Christensenellaceae_R-7_group*, *Lactobacillus*, *Turicibacter, norank_f_norank_o_Clostridia_UCG-014*	

*^a^*MRA, means relative abundance. Note: Neutralists were not list in the table.

### 3.4 Microbial pathway regulation

**FIGURE 6:**
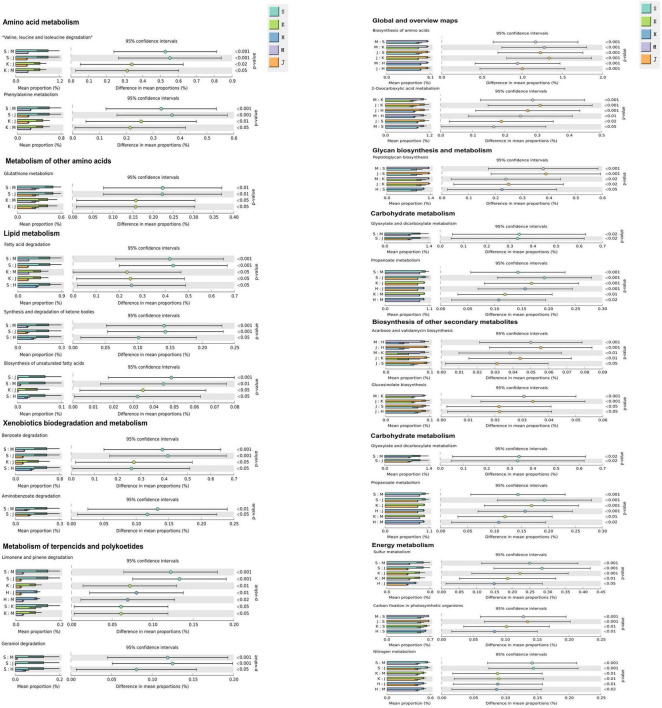
The bar graph shows the microbial functions predicted using PICRUSt2 at the third level of the KEGG pathway for the swine gut microbiome. S, Duodenum; K, Jejunum; H, Ileum; M, Cecum; J, Colon. The “S:K” ratio represents the comparison between duodenal and jejunal microbial functions; the meanings of the other corresponding ratios are adjusted according to their respective letters.

The KEGG metabolic pathways were predicted for the gut microorganisms across in different intestinal segments in swine (Figure 6). These metabolic pathways include Lipid metabolism (Fatty acid degradation), Amino acid metabolism (Valine, leucine, and isoleucine degradation and Phenylalanine metabolism), Metabolism of other amino acids (Glutathione metabolism), Metabolism of terpencids and polykoetides (Limonene and pinene degradation), Carbohydrate metabolism (Propanoate metabolism), and Sulfur and Nitrogen metabolism, all of which were significantly higher in the duodenum and jejunum (*p* < 0.05) when compared to the cecum and colon. Additionally, Lipid metabolism (Synthesis and degradation of ketone bodies and Biosynthesis of unsaturated fatty acids), Xenobiotics biodegradation and metabolism (Degradation of Benzoate and Aminobenzoate), Metabolism of terpencids and polykoetides (Geraniol degradation), Carbohydrate metabolism (Glyoxylate and dicarboxylate metabolism) in the KEGG pathways were significantly elevated in the duodenum (*p* < 0.05). Moreover, Metabolism of terpencids and polykoetides (Limonene and pinene degradation), Carbohydrate metabolism (Propanoate metabolism), and Nitrogen metabolism were significantly higher in the ileum (*p* < 0.05). Global and overview maps (2-Oxocarboxylic acid metabolism and Biosynthesis of unsaturated fatty acids) were significantly enhanced in the cecum and colon compared to the duodenum, jejunum, and ileum (*p* < 0.05). Energy metabolism (Carbon fixation in photosynthetic organisms)and Glycan biosynthesis and metabolism (Peptidoglycan biosynthesis) pathways were both significantly elevated in the cecum and colon (*p* < 0.05) relative to the duodenum. Additionally, Biosynthesis of other secondary metabolites (i.e., Acarbose and validamycin biosynthesis, Glucosinolate biosynthesis) and Glycan biosynthesis and metabolism (Peptidoglycan biosynthesis) pathways showed significantly higher in the cecum and colon compared to the jejunum (*p* < 0.05). Compared to the ileum, Acarbose and validamycin biosynthesis, which belong to the Biosynthesis of other secondary metabolites pathway, was significantly elevated in the cecum and colon (*p* < 0.05).

## 4 Discussion

### 4.1 Diversity and structure of gut microbial biogeography in swine

The diversity analysis revealed significant differences in microbial community composition between the small intestine (duodenum, jejunum, ileum) and large intestine (cecum, colon). The microbial community structure in the cecum and colon was more complex, exhibiting greater richness and diversity compared to the duodenum, jejunum, and ileum as reported in the previous studies on intestinal microbial communities (Yang et al., 2016; Yang et al., 2020). The distribution pattern of intestinal microbial communities may be linked to functional disparities between the small and large intestines. The small intestine as a primary site for nutrient absorption (monosaccharides, proteins, lipids) owing to its physiological characteristics including lower pH, shorter flow, reduced peristalsis rates, and a facultative anaerobic environment, which collectively render it less conducive to high microbial diversity and abundance (Donaldson et al., 2016; Sheth et al., 2019). In contrast, the large intestine features a neutral pH and greater quantities of undigested starches, unabsorbed sugars, and polysaccharides derived from the small intestine, factors that favor a higher abundance and diversity of bacterial taxa (Hillman et al., 2017; Zong et al., 2024).

### 4.2 Gut microbial biogeography in swine

In this study, the core phyla included Firmicutes (32.5–90.4%), Proteobacteria (0.503–34.2%), Actinobacteriota (0.704–23.3%), and Bacteroidetes (0.137–7.68%). This differs from previous studies on intestinal microbes in pigs. A meta-analysis from 20 studies indicated that Firmicutes, Proteobacteria, and Bacteroidetes were the predominant phyla, collectively representing over 90% relative abundance throughout the entire gastrointestinal tract in swine (Holman et al., 2017). However, the relative abundance and the proportions of phyla are strongly correlated with host factors such as obesity, diet, age, and breed (Kim et al., 2011; Pajarillo et al., 2015; Zhao et al., 2015; John and Mullin, 2016; Yang et al., 2018; Rinninella et al., 2019). A study identified Firmicutes, Actinobacteriota, Bacteroidetes, and Proteobacteria as core phyla in the gut of wild pigs (*Sus scrofa ussuricus*) (Yang et al., 2020). We also observed differences in the relative abundance of these phyla across intestinal segments. The phylum Actinobacteria was dominant in the duodenum (23.3%) and exhibited significantly higher abundances in the cecum and colon (*p* < 0.001). Moreover, LEfSe analysis also indicated the enrichment of aerobic or parthenogenetic anaerobes (*Corynebacterium*, *Dietzia*, *Rhodococcus*) within the phylum Actinobacteria, consistent with the previous studies (Friedman et al., 2018). Recent studies have reported that the phylum Actinobacteria is involved in the synthesis of immunomodulatory compounds, key antibiotics, and metabolites in the animal gut that are critical to host health and homeostasis (Matsui et al., 2012; Rodríguez-Sorrento et al., 2021). Although studies have shown that pig intestinal microorganisms are affected by breed, gender and age, diet is the main concern that shaping the intestinal microorganism profiles (Duarte and Kim, 2022). Zhao et al. (2015) have reported that the gut microbiota are relatively stable at 6 months of age. In this study, intestinal microorganisms tended to be relatively stable. For the research or determination of pig intestinal microorganisms, it is recommended to collect samples from at least 5 to 6 pigs for each research or testing project. Many previous studies have also shown that this recommendation helps ensure that the microbiological data obtained have sufficient statistical significance and a broad representative range (Nair et al., 2019; Shao et al., 2021; Qi et al., 2022; Zhu et al., 2022). Thus, this study mainly provides basic research guidance for analyzing the geographical distribution of intestinal microorganisms in grow-finishing pigs of ordinary feeding conditions.

We also recorded a higher abundance of phylum Proteobacteria in the duodenum (34.2%) and jejunum (10.4%), which aligns with findings from other studies (Zhao et al., 2015). Proteobacteria is reported to be the second most abundant phylum in the duodenum (Li et al., 2023), which is consistent with our findings. This phylum exhibits a high tolerance to the unfavorable environmental conditions of the small intestine (Angelakis et al., 2015; Leone et al., 2015). Furthermore, Proteobacteria facilitate microbial colonization in the duodenum by consuming oxygen and contribute to the development of the host’s immune system (Gomez et al., 2013; Rey et al., 2014). We observed a significantly higher abundance of phylum Proteobacteria in the jejunum through LEfSe analysis, consistent with previous studies (Leite et al., 2020). In this study, the phyla Firmicutes (90.4%) and Bacteroidetes (7.06%) were dominant in the colon, as reported in previous studies (Xiao et al., 2018; Huang et al., 2024). The phylum Firmicutes is known for its ability to degrade fibrous materials by breaking down cellulose into volatile fatty acids for host utilization, thereby enhancing nutrient absorption and modulating T cells to strengthen immune response and prevent intestinal inflammation. Meanwhile, phylum Bacteroidetes play a crucial role in degrading and assimilating polysaccharides, carbohydrates, and proteins in the gut (Guan et al., 2017; Zhang L. et al., 2018; Zafar and Saier, 2021). Notably, despite the high abundance of Firmicutes in both the small and large intestines (32.5–90.4%), its composition differs between these regions in our study.

The small intestine was predominantly occupied by facultative anaerobes, such as *Corynebacterium*, *Peptostreptococcus*, and *Escherichia-Shigella*, while the large intestine was dominated by specialized anaerobes, such as *Akkermansia*, *Christensenellaceae R-7_group*, and *Ruminococcus*. In this study, we observed distinct dominant genera in various intestinal segments of swine. *Fusobacterium* was found to be the dominant microbiota during the grower-finisher period. Such a genus may help reduce the risk of infectious intestinal diseases and ensure the growth of the host (Luo et al., 2022). The genus *Corynebacterium* is critical to the swine industry and has shown a positive association with porcine feed efficiency (McCormack et al., 2017). The higher abundance of *Rothia* (5.71%) was observed in the duodenum, while it was nearly absent in the cecum and colon. *Rothia* has been identified as a dominant flora in the upper respiratory tract and stomach (Bik et al., 2006; Engstrand and Lindberg, 2013). This finding also suggests that some of the bacteria present in the duodenum may originate from the microbes found in the oral cavity and stomach regions. *Bradyrhizobium* was positively correlated with crude protein and could synthesize proteins (through nitrogen fixation) and lipids (Cui et al., 2024). The *Brevundimonas* include cellulolytic and xylanolytic strains, which were identified in the gastrointestinal tract of young pigs (Motta et al., 2017). A significantly higher abundance of genus *Romboutsia* was found in the jejunum (36.1%) and ileum (31.1%), consistent with previous studies (Gerritsen et al., 2014; Gerritsen et al., 2017; Quan et al., 2018). Additionally, the genus *Clostridium_sensu_stricto_1* displayed higher relative abundance in the jejunum (18.6%), corroborating earlier research (Crespo-Piazuelo et al., 2018). We recorded a higher abundance of *Turicibacter* (3.86%) and *Terrisporobacter* (5.72%) in the ileum, consistent with prior studies (Looft et al., 2014; Quan et al., 2018). In our study, *Oscillospiraceae_UCG-005* (16.4%), *norank_f_Eubacterium_coprostanoligenes_group* (8.28%), and *Clostridium_sensu_stricto_1* (7.62%) were identified as the predominant bacterial genera in the cecum of swine. A comparative study indicated a higher relative abundance of *Oscillospiraceae_UCG-005* in swine fed a low-protein diet compared to those on a normal protein diet (Liao et al., 2024). *Akkermansia* was found to be linked with better feed efficiency, metabolic disorders, and intestinal inflammation. It can be an indicator of healthier intestinal function and is related to mucin degradation (McCormack et al., 2017). In the colon, *Christensenellaceae_R-7_group* (20.2%), *Clostridium_sensu_stricto_1* (9.55%), and *Lactobacillus* (5.62%) were the dominant genera, these findings echoed in a study involving 240-day-old Landrace and Jinhua swine (Xiao et al., 2018). Another study on 140-day-old swine reported an abundance of *Lactobacillus* and *Clostridium* in the colon (Quan et al., 2018). The abundance of *Ruminococcaceae UCG-013* and *Christensenellaceae R-7 group* in the colon was also reported in pigs (Song et al., 2022). With regard to the microbes in different intestinal segments, the key microbes need to be highlighted and further explored.

### 4.3 Microbial functional niche and their pathways

Microorganisms enhance their survival capabilities by adapting as either generalists (which can survive in diverse habitats) or specialists (which are more adapted to specific habitats) (Van Tienderen, 1991). In our study, we observed a higher number of specialists compared to generalists at the genus level across various intestinal segments, consistent with prior research (Sriswasdi et al., 2017; Yang et al., 2023).This might be attributed to the transient state of the microbes becoming generalists through evolution while most microbes eventually evolved into specialists after undergoing certain evolutionary pressures (Sriswasdi et al., 2017; Xu et al., 2022a). Our findings indicate that both generalists and specialists are more prevalent in the small intestine than in the large intestine. A possible explanation is that the small intestine is a more heterogeneous environment than the large intestine, leading to greater unknown environmental sources and increased environmental filtering. Consequently, in order to cope with the constant movement and competition from invasive species, microorganisms enhance their survival capabilities by becoming generalists or specialists (Nagelkerke and Menken, 2013). Moreover, generalists can thrive in various habitats and may exhibit a large fundamental metabolic niche characterized by high metabolic plasticity. They also gain a survival advantage by exploiting unused habitats of specialists and demonstrating greater functional plasticity, playing a crucial role in the formation of new species and the maintenance of biodiversity (Sriswasdi et al., 2017; Muller, 2019). Therefore, it is inferred that the microbiome in the small intestine is more flexible in terms of metabolism, capable of adapting to a broader range of environments, and is better able to resist environmental influences than the microbiome in the large intestine, playing an important role in maintaining stability in the small intestine. Finally, our results regarding the classification of specialists indicate that there are differences in specialists across different intestinal segments, which may be related to the distinct microenvironmental characteristics in the gut (e.g., oxygen concentration, pH, and metabolites). Compared to the large intestine, the small intestine has shorter transit times, lower pH levels, and higher concentrations of oxygen and antimicrobial peptides (AMPs) (McCallum and Tropini, 2024). Consequently, the small intestine predominantly hosts rapidly proliferating facultative anaerobic bacteria, such as *Streptococcus*, *Escherichia-Shigella*, and *Pseudomonas*. In contrast, the large intestine primarily harbors a diverse array of sugar-degrading anaerobic bacteria, including *Akkermansia*, *Oscillospiraceae UCG-005*, *unclassified_f_Lachnospiraceae*, and *Christensenellaceae_R-7_group*.

In the small intestine, our study revealed the enrichment of microorganisms in the duodenum, jejunum, and ileum of swine in pathways related to Amino acid and Lipid metabolism. Our findings indicated that *Romboutsia* and *Clostridium sensu stricto 1* were specialists in the duodenum and jejunum, while *Terrisporobacter* was a specialist in the jejunum. Additionally, *Clostridium sensu stricto 1* and *Romboutsia* predominated in the duodenum, jejunum, and ileum, alongside *Turicibacter* and *Terrisporobacter* in the jejunum and ileum. These microbes are likely associated with their corresponding metabolic pathways. The genus *Romboutsia* has been shown to possess the capacity for carbohydrate absorption and amino acid fermentation, thereby contributing to host health (Ricaboni et al., 2016; Mangifesta et al., 2018). *Clostridium* is among the representatives of intestinal commensal bacteria possessing the potent probiotic characteristics to maintain intestinal homeostasis (Guo et al., 2020). This genus can digest a variety of nutrients, including carbohydrates, proteins, organic acids, and other organic materials, to produce primary short-chain fatty acids such as acetate, propionate, and butyrate, as well as some solvents in the jejunum and ileum (Ricaboni et al., 2016). Furthermore, numerous studies have reported a correlation between *Turicibacter* and host fat metabolism (Petersen et al., 2019; Dhakal et al., 2020), as well as an association between the abundance of *Terrisporobacter* and triglyceride metabolism related to C-reactive protein (Lee et al., 2020).

In the large intestine, microbial communities in the cecum and colon exhibited significant enrichment in Glycan biosynthesis and metabolism, as well as the Biosynthesis of other secondary metabolites. This enrichment may be attributed to the abundance of specific microbes, such as *Oscillospiraceae_UCG-005* and *Christensenellaceae R-7 group*, found in the cecum and colon, respectively. Additionally, these genera were also identified as specialists in the cecum and colon during our analysis. The cecum and colon serve as a crucial site where indigestible carbohydrates such as fiber are fermented and metabolized by the microorganisms to generate bioavailable nutrients. The Oscillospiraceae represents a microbial consortium specialized in the degradation of complex substrates and synthesis of short-chain fatty acids (Vedel et al., 2023). Recent studies have also demonstrated the beneficial impact of *Oscillospiraceae_UCG-005* on the intestinal health of animals (Konikoff and Gophna, 2016; Xu et al., 2022b). Christensenellaceae has been reported to produce butyrate, which not only serves as the primary energy source for colonic epithelial cells (Sun et al., 2019; Liu et al., 2021) but also contributes to the degradation of plant polysaccharides (Vital et al., 2014). Moreover, the *Christensenellaceae_R-7_group*, a dominant genus within the Christensenellaceae family, is widely distributed in the colon and is considered a potential probiotic for promoting host health (Waters and Ley, 2019).

## 5 Conclusion

This study revealed that the microbial composition and abundance in five intestinal segments of pigs are structured differently, reflecting the functional adaptation of each intestinal region. This variation provides evidence of biogeographic stratification of the microbial community across different spatial scales within the intestine. We found Firmicutes (32.5–90.4%), Proteobacteria (0.503–34.2%), Actinobacteriota (0.704–23.3%), Fusobacteriota (0.018–7.68%), and Bacteroidetes (0.137–7.06%) were the dominant phyla collectively accounting for over 90% of the total relative abundance. At the genus level, *Bradyrhizobium* (0–5.24%), *Romboutsia* (3.06–36.1%), *Clostridium_sensu_stricto_1* (5.31–18.6%), and *Terrisporobacter* (0.849–5.72%) emerged as the dominant genera and specialists associated with Amino acid and Lipid metabolic pathways in the small intestine. In the cecum and colon, *Oscillospiraceae_UCG-005* (16.4%) and *Christensenellaceae_R-7_group* (20.2%) were identified as dominant genera and specialists, respectively, linked to Glycan biosynthesis and metabolism pathway, and Biosynthesis of other secondary metabolites pathway. Additionally, we observed a greater abundance of specialists than generalists in this study, with generalists in the small intestine being more numerous than those in the large intestine of swine. These findings provide fundamental data for evaluating the intestinal microbial community and offer essential guidance for further exploration of host-microbe interaction mechanisms that support the health and productivity of pigs.

## Data Availability

The unprocessed 16S rRNA sequence data reported is available in the NCBI Sequence Read Archive (SRA) database under the BioProject ID: PRJNA1183991.

## Footnotes

1
https://ccb.jhu.edu/software/FLASH/

2
https://rrndb.umms.med.umich.edu/estimate/

3
http://huttenhower.sph.harvard.edu/lefse
