# Supplementary material for: Survey of gut microbial biogeography and their functional niche in the grow-finishing swine of ordinary feeding
Source: Front Microbiol. 2025 Mar 7;16:1530553. doi: 10.3389/fmicb.2025.1530553 (PMC11925874; doi:10.3389/fmicb.2025.1530553)
Supplement: Supplementary file 1 [file Table_1.docx]

**Table S1** Ingredients and composition of the basal diet (air-dried)

| **Items** | **Content, %** |
| --- | --- |
| **Ingredients** |  |
| Corn | 46.0 |
| Wheat | 32.3 |
| Soybean meal | 19.0 |
| CaHPO_4_ | 1.00 |
| NaCl | 0.30 |
| Limestone | 0.90 |
| Premix^1^ | 0.50 |
| Total | 100 |

^1^The premix provides the following per kg of the diet: VA 6,200 IU, VB_1_ 1.6 mg,VB_2_ 5 mg,VB_6_ 5 mg,VB_12_ 0.022 mg, VD_3_ 3,100 IU, VE 50 IU, VK_3_ 2.5 mg, biotin 4.0 mg, folic acid 0.4 mg, niacin 23 mg, pantothenic acid 21 mg, choline 500 mg, Cu (CuSO_4_·5H_2_O) 14 mg, Fe (FeSO_4_) 95 mg, Mn (MnSO_4_·H_2_O) 95 mg, Zn (ZnSO_4_) 50 mg, I (KI) 0.5 mg, Se (Na_2_SeO_3_) 0.2 mg.
